# Genetic susceptibility to kidney stone disease: unveiling pathogenesis and potential therapeutic targets

**DOI:** 10.1172/JCI195624

**Published:** 2025-08-01

**Authors:** Shiwei Li, Xuemei Wang, Ming Liu

**Affiliations:** 1 Department of Endocrinology and Metabolism, Tianjin Medical University General Hospital, Tianjin, China.; 2 Department of Endocrinology and Nephrology, Tianjin Ninghe Hospital, Tianjin, China.

## Abstract

Kidney stone disease (KSD) arises from a complex interplay of genetic predisposition, diet, metabolic disorders, and other environmental factors. In this issue of the *JCI*, Lovegrove et al. report a large GWAS that identifies 71 loci associated with an increased risk of KSD. Through an integrative approach combining Mendelian randomization and functional validation, they emphasize the roles of *DGKD*, *SLC34A1*, and *CYP24A1* in maintaining homeostasis of calcium and phosphate. These findings offer insights into the pathogenesis of KSD and suggest potential targets for intervention. Further studies are needed to validate these findings across diverse populations and clinical settings.

## The genetic basis of kidney stone disease

Kidney stone disease (KSD) is a common and recurrent urological condition that affects approximately 10% of the global population and imposes a substantial burden on health care systems (1). Although the acute pain from urinary stone passage is well recognized, the underlying pathogenesis of stone formation and its chronic consequences, including genetic predispositions, systemic metabolic disturbances, recurrent stones, and renal dysfunction, remain less understood (2). The substantial economic burden and patient morbidity underscore the need to elucidate the underlying pathophysiology (3). While modifiable factors such as diet, fluid intake, metabolic disorders, infections, and certain drugs have traditionally been the focus of prevention strategies (4, 5), growing evidence points to a critical role for inherited susceptibility.

Twin studies have shown that the heritability of KSD may exceed 50% (6), and family history remains one of the strongest clinical predictors of KSD (7). These familial patterns suggest that genetic variants influence renal handling of calcium, phosphate, oxalate, and uric acid. Indeed, rare monogenic disorders including X-linked Dent disease and mutations in *SLC34A3* impair proximal tubule (PT) function and phosphate reabsorption, respectively, leading to nephrocalcinosis and an increased risk of stone formation. Inherited hyperparathyroidism, as seen in multiple endocrine neoplasia (MEN) syndromes, results in hypercalcemia and hypercalciuria, further promoting lithogenesis (8). Although individual loci associated with these rare causes of KSD have provided insights into disease mechanisms, their translation into diagnostic or therapeutic strategies in the general population remains elusive.

Initial GWAS identified several risk loci, including *CLDN14*, *SLC34A1*, and *TRPV5* (1, 9, 10), implicating pathways involved in mineral reabsorption and epithelial transport. However, the predominance of Icelandic and Japanese cohorts in these studies limits the generalizability of the findings to more diverse populations (2). In this issue of the *JCI*, Lovegrove et al. report an important advance (11). By integrating GWAS with Mendelian randomization (MR), genetic colocalization, and in vitro functional studies, the authors provide a robust framework linking genetic variation to mineral homeostasis, calcium-sensing receptor (CaSR) signaling, and clinical phenotype.

## From genetic variants to targeted interventions

**Figure 1:**
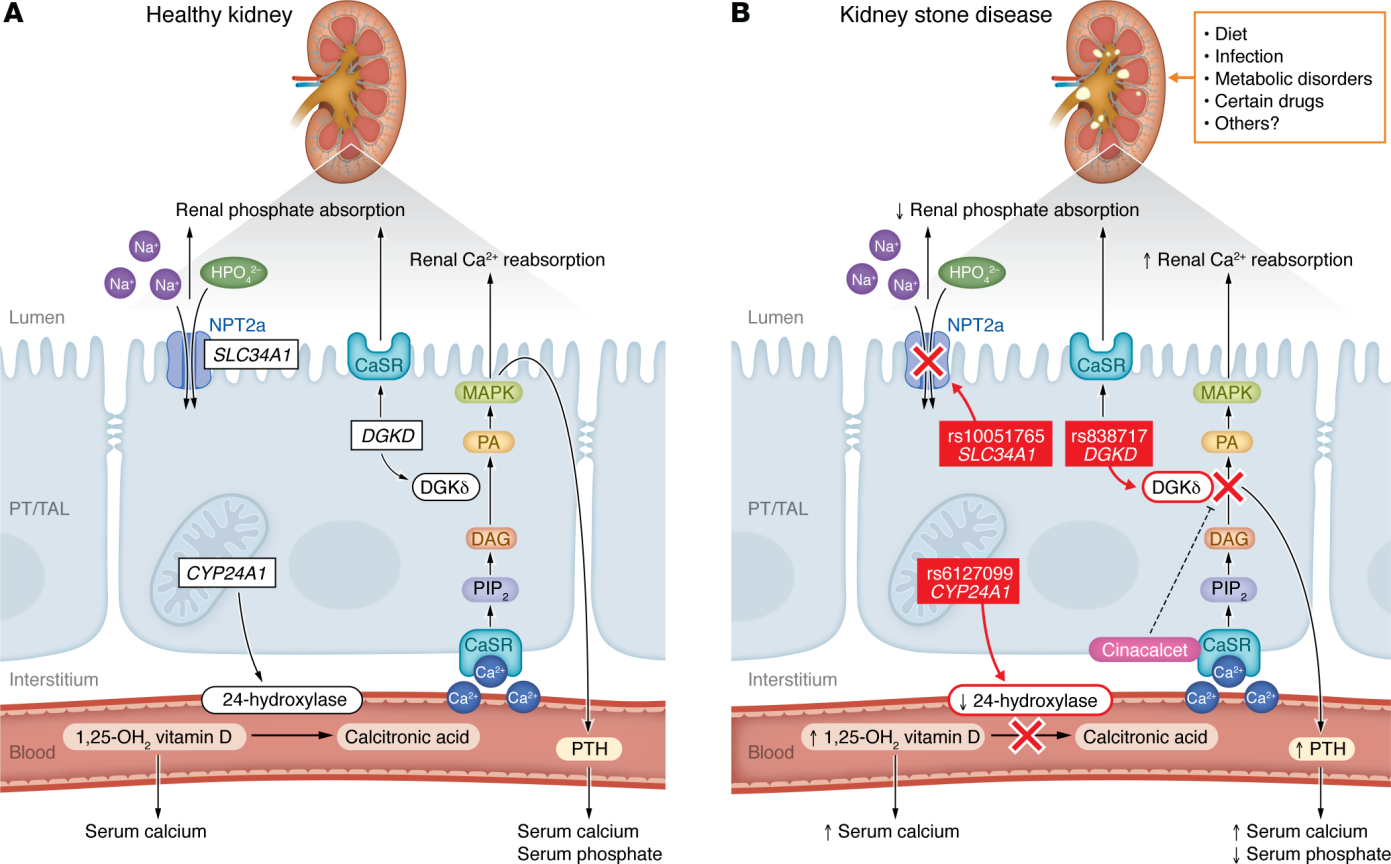
Genetic variants in *DGKD*, *SLC34A1*, and *CYP24A1* and environmental factors contribute to the pathogenesis of KSD. (**A**) The healthy kidney maintains homeostasis of calcium and phosphate. (**B**) Several variants independently perturb the calcium-phosphate balance and are associated with increased susceptibility to KSD. The *DGKD* variant rs838717 impairs CaSR signaling via DGKδ activity, disrupting conversion of DAG to PA, a lipid mediator downstream of MAPK signaling that modulates calcium and phosphate homeostasis. This defective signaling increases calcium reabsorption in the TAL and augments phosphate excretion in the PT. Moreover, rs838717 may contribute to elevated circulating calcium and reduced phosphate levels by directly promoting PTH secretion. Cinacalcet can partially rescue the impaired CaSR signal transduction. The *SLC34A1* variant rs10051765 impairs phosphate reabsorption by reducing the activity of sodium-phosphate cotransporter NPT2a in the PT, leading to increased renal phosphate excretion. The *CYP24A1* variant rs6127099 decreases 24-hydroxylase activity, resulting in elevated circulating 1,25-dihydroxyvitamin D and increased renal calcium absorption. Environmental and acquired risk factors, including dietary oxalate, sodium, animal protein intake, urinary tract infection, medications, metabolic disorders, and unidentified contributors, interact with genetic susceptibility to promote lithogenesis. MAPK, mitogen-activated protein kinase; PA, phosphatidic acid; PIP_2_, phosphatidylinositol 4,5-bisphosphate.

Lovegrove and colleagues conducted one of the most comprehensive genetic studies of KSD to date, integrating GWAS data from more than 1.2 million individuals with MR, as well as data from colocalization, drug-target MR, and in vitro functional assays (11). They identified 79 independent risk signals across 71 genomic loci, markedly expanding the known genetic landscape of KSD. A key strength of this work lies in its translational focus. Rather than limiting the analysis to statistical associations, the authors followed a gene-pathway-therapy framework, exemplified by three key candidate genes: *DGKD*, *SLC34A1*, and *CYP24A1* (Figure 1) (11).

*DGKD* encodes a diacylglycerol (DAG) kinase that modulates CaSR signaling. Functional studies have shown that the KSD-associated variant rs838717 impairs CaSR-mediated activation of MAPK pathways, resulting in decreased phosphate reabsorption in the PT and increased calcium reabsorption in the thick ascending limb (TAL) of the loop of Henle (11, 12). The authors showed this variant may also enhance parathyroid hormone (PTH) secretion, contributing to elevated serum calcium and reduced serum phosphate (11). Through direct effects on TAL calcium transport and indirect effects via PTH-mediated inhibition of proximal phosphate reabsorption, the rs838717 variant may disrupt homeostasis of calcium and phosphate and promote lithogenesis (11). *SLC34A1* encodes the sodium-phosphate cotransporter NPT2a, which is essential for phosphate reabsorption in the renal PT. Lovegrove and co-authors found that the variant rs10051765 in *SLC34A1* was associated with increased urinary phosphate, potentially leading to calcium phosphate supersaturation and promoting stone formation (11). *CYP24A1* encodes the enzyme 24-hydroxylase, which is responsible for metabolizing active vitamin D (1,25-dihydroxyvitamin D). The variant rs6127099 in *CYP24A1* was associated with reduced 24-hydroxylase activity, resulting in elevated 1,25-dihydroxyvitamin D_3_ and subsequently contributing to increasing serum calcium, which occurs in conditions linked to increased KSD (11).

MR and colocalization analyses have been used to infer causal relationships between imbalances in serum minerals and the risk of KSD (13–15). By leveraging independent genetic variants as instrumental variables, Lovegrove et al. present evidence that even modest, genetically mediated changes in serum calcium or phosphate levels can substantially alter the risk of kidney stone formation. Specially, MR analysis suggests that a one SD increase in serum calcium mediated via *DGKD* variants confers more than a four-fold increase in KSD risk (11). These mechanistic hypotheses are further supported by colocalization and expression quantitative trait locus (eQTL) analyses and are reinforced by MR studies that suggest a causal link between gene expression levels and KSD risk. While these findings require cautious interpretation due to the complexity of calcium homeostasis, they provide a framework for understanding how common genetic variation cumulatively influences disease susceptibility (11).

Notably, the work by Lovegrove et al. parallels and complements a contemporaneous trans-ancestry GWAS by Cao et al., recently published in *Nature Communications* (16), which aimed to elucidate the genetic architecture of KSD. Methodologically, Cao et al. conducted cross-population GWAS meta-analyses and validated polygenic risk scores (PRSs) across multiple ancestries, improving generalizability but introducing potential limitations related to diagnostic heterogeneity and differences in linkage disequilibrium structure (16). In contrast, Lovegrove et al. focused primarily on European ancestry cohorts, which limits ethnic generalizability but offers greater mechanistic depth through MR, colocalization, and functional validation (11). These two studies considerably expanded the list of KSD-associated loci and identified shared genes, including *SLC34A1* and *CYP24A1*. However, each study also uncovered distinct associations: Lovegrove et al. highlighted variants in *DGKD* (11), whereas Cao et al. identified novel loci such as *TRIOBP* by leveraging fine-mapping across European and East Asian populations (16). These complementary approaches underscore the value of integrating population-scale discovery with functional genomics to advance the understanding of KSD.

Beyond mechanistic insights, drug-target MR simulations that modeled the effects of enhancing or inhibiting activity at these risk loci provide a preclinical rationale for therapeutic development. Importantly, these analyses indicate that modulation of serum calcium via *CASR*, *DGKD*, or *CYP24A1*, or increased phosphate reabsorption via *SLC34A1*, could reduce the risk of KSD by as much as 90% (10). Specifically, cinacalcet, a clinically approved CaSR agonist (17), partially rescued impaired CaSR signaling caused by *DGKD* variants in genetically susceptible individuals (11). Similarly, targeting the vitamin D pathway, for example, through inhibitors of vitamin D synthesis, may be beneficial in individuals carrying high-risk *CYP24A1* variants (10). These scenarios underscore the translational potential of the gene-pathway-therapy paradigm and may promote a shift from conventional management, which has focused largely on symptom control and general lifestyle modifications, to more targeted intervention based on genetic susceptibilities.

## Conclusions and future perspectives

Lovegrove et al. provide a foundation for several future directions in the genetic and clinical management of KSD. The expanded list of risk loci enables the construction of a more precise PRS, which could identify individuals at increased risk for incidents or recurrent stones. The identification of druggable targets, including diacylglycerol kinase δ (DGKδ) and components of the CaSR signaling pathway, supports the potential repurposing of existing medications for genetically susceptible individuals. These genotype-guided approaches could serve as the basis for future randomized clinical trials. However, continued validation in ancestrally diverse cohorts is essential. Broader representation will be essential for equitable application of genomic tools (18).

Looking ahead, several key questions remain. First, how can we better quantify the individual and cumulative contributions of genetic risk alleles to disease burden? Future efforts should focus on refining PRSs across ancestries and integrating them with clinical and biochemical data to enhance risk stratification, particularly in early-onset or idiopathic cases (19). Second, what molecular mechanisms underlie polygenic interactions that influence stone formation? Elucidating these interactions will require integrative multiomics approaches and high-throughput functional assays to reveal how combinations of variants affect mineral handling and epithelial transport. Third, how can these findings be translated into effective interventions? One direction involves genotype-guided trials evaluating repurposed therapies, while another lies in developing risk-stratified lifestyle modification protocols. Finally, how do environmental and lifestyle exposures interact with genetic predisposition to influence stone formation? Understanding gene-environment interplay, such as sodium and calcium intake in individuals with altered CaSR signaling, may inform targeted prevention strategies and increase the clinical utility of genetic screening. Advancing this agenda will require close interdisciplinary collaboration among geneticists, endocrinologists, nephrologists, and nutritionists. By combining genomic, clinical, and behavioral data, future research may enable precision prevention strategies and improved long-term outcomes for individuals at risk of KSD (20).

## Version 1 08/01/2025

Electronic publication
